# Proof of the Concept to Use a Malignant B Cell Line Drug Screen Strategy for Identification and Weight of Melphalan Resistance Genes in Multiple Myeloma

**DOI:** 10.1371/journal.pone.0083252

**Published:** 2013-12-20

**Authors:** Martin Bøgsted, Anders E. Bilgrau, Christopher P. Wardell, Uta Bertsch, Alexander Schmitz, Julie S. Bødker, Malene K. Kjeldsen, Hartmut Goldschmidt, Gareth J. Morgan, Karen Dybkaer, Hans E. Johnsen

**Affiliations:** 1 Department of Haematology, Aalborg Hospital Science and Innovation Center (AHSIC), Aalborg University Hospital, Aalborg, Denmark; 2 Haemato-Oncology Research Unit, Division of Molecular Pathology, Institute of Cancer Research, London, United Kingdom; 3 Department of Internal Medicine V and National Center for Tumor Diseases, University of Heidelberg, Heidelberg, Germany; 4 Department of Mathematical Sciences, Aalborg University, Aalborg, Denmark; University of Birmingham, United Kingdom

## Abstract

In a conceptual study of drug resistance we have used a preclinical model of malignant B-cell lines by combining drug induced growth inhibition and gene expression profiling. In the current report a melphalan resistance profile of 19 genes were weighted by microarray data from the MRC Myeloma IX trial and time to progression following high dose melphalan, to generate an individual melphalan resistance index. The resistance index was subsequently validated in the HOVON65/GMMG-HD4 trial data set to prove the concept. Biologically, the assigned resistance indices were differentially distributed among translocations and cyclin D expression classes. Clinically, the 25% most melphalan resistant, the intermediate 50% and the 25% most sensitive patients had a median progression free survival of 18, 32 and 28 months, respectively (log-rank P-value  = 0.05). Furthermore, the median overall survival was 45 months for the resistant group and not reached for the intermediate and sensitive groups (log-rank P-value  = 0.003) following 38 months median observation. In a multivariate analysis, correcting for age, sex and ISS-staging, we found a high resistance index to be an independent variable associated with inferior progression free survival and overall survival. This study provides clinical proof of concept to use *in vitro* drug screen for identification of melphalan resistance gene signatures for future functional analysis.

## Introduction

Multiple Myeloma (MM) is an incurable B-cell malignancy that ultimately relapses due to resistant disease despite advances in therapeutic approaches [1], [2]. Improved molecular profiling technologies [3] have advanced the pathogenetic understanding [4] and introduced the concept of targeted therapy, challenging existing strategies. The transition from the long-established one-size-fits all approach to new strategies, based on individual genetic and gene expression profiles, provides an opportunity to transform current diagnostics into individual prognostic or even predictive classifications.

The ultimate goal for an individualized treatment strategy is to have diagnostic tests predicting drug specific resistance. However, this is currently not state of the art in MM where a number of prognostic systems exist. The most commonly used is the international staging system (ISS) based on clinical features [5]. It has been shown that ISS can be improved by the integration of cytogenetic findings, which are independently associated with poor prognosis [6]–[10]. These observations underline the importance of genetic biomarkers in determining the optimal treatment approach in MM, and constitute the first step towards a personalised treatment approach, but do not constitute true prediction of the individual response to a single drug.

Different mechanisms of resistance to therapy have been described; first, intrinsic genetic resistance associated with the t(4;14), t(14;16), t(14;20) or the presence of 17p deletion; secondly, acquired resistance upon treatment; thirdly, cell adhesion mediated drug resistance (CAMDR) and finally, inherited genetic variation. Understanding the mechanisms at a molecular level remains a pivotal issue, requiring biological models and global expression profiling, gene mapping, methylation mapping, mutation detection and miRNA assays for biomarker discovery.

It is our concept that malignant B-cell lines can be used as a preclinical model for B-cell malignancies as they have evolved from intrinsic and acquired genetic events, and therefore harbour the most extensive molecular mechanisms of resistance. The availability of these cell lines may accelerate the therapeutic advancements towards individualised therapy and we have recently suggested a list of 19 genes with potential impact on resistance to melphalan treatment based on an *in vitro* drug screen set up mimicking the NCI60 cell line based screening platform [11], [12]. Similar studies have been published for cell lines derived from breast and lung cancer [13], [14].

In the current study we have addressed the approach presented by Lee and co-workers [15], adjusting the aforementioned *in vitro* cell line based drug resistance gene list to the range of molecular expression in newly diagnosed tumours. The outcome of such a strategy may be an improved patient weighted gene index predictive for melphalan resistance. Importantly, such a resistance index should be validated in an independent set of clinical studies to support the above mentioned concept. The data sets used in this analysis are derived from the recently published HOVON65/GMMG-HD4 trial [16] and MRC Myeloma IX trial [17].

A successful validation of our strategy will allow us to select genes that may be involved in the molecular mechanisms of resistance and perform biological or functional studies. Ultimately, such results may identify a panel of potential genes and reverse translate these into a predictive diagnostic platform for prospective studies.

## Patients, Materials and Methods

Retrospective data from newly diagnosed MM patients including clinical characteristics, follow-up data and diagnostic global gene expression profile (GEP) analysis were available for 263 patients entering the HOVON65/GMMG-HD4 [16] and 94 patients from Royal Marsden Hospital London entering the MRC Myeloma IX [17] clinical trials approved by the local institutional review boards as stated in references 16 and 17. Both clinical trials were randomized multicenter studies comparing different induction chemotherapy regimens prior to high dose melphalan (HDM) and both trials included maintenance therapy randomizations. Patients were selected for diagnostic global GEP analysis on plasma cells and a set of classical prognostic and clinical outcome variables were recorded. The characteristics of the two independent trials, including median follow up time, are given in Table 1.

**Table 1:** Characterisation of the clinical trial data sets.

Patient Characteristics	HOVON65/GMMG-HD4	MRC Myeloma IX	P-value
n	263	94	
**Age**			0.023
Median	56	57	
Range	(27,65)	(35,69)	
**Sex**			0.62
Female	116 (44%)	38 (40%)	
Male	147 (56%)	56 (60%)	
**ISS**			0.46
I	96 (39%)	24 (32%)	
II	88 (36%)	30 (39%)	
III	60 (25%)	22 (29%)	
**TC class**			0.54
11q13	34 (13%)	16 (17%)	
4p16	39 (15%)	20 (21%)	
6p21	4 (2%)	1 (1%)	
D1	103 (39%)	36 (38%)	
D1plusD2	7 (3%)	2 (2%)	
D2	35 (13%)	6 (6%)	
MAF	12 (5%)	3 (3%)	
none	29 (11%)	10 (11%)	
**Follow up time**			
median	37.98	49.5	
**Survival**			
median OS	NA	NA	
(0.95LCL,0.95UCL) OS	(NA,NA)	(51.8,NA)	
median PFS	26.7	23.5	
(0.95LCL,0.95UCL) PFS	(22.8,31.5)	(17.8,30.3)	
**Treatment**			
	VAD: 124 (47%)	CVAD: 38 (40%)	
	PAD: 139 (53%)	CTD: 56 (60%)	

Comparison of the two independent HOVON65/GMMG-HD4 and MRC Myeloma IX cohorts with respect to demographic data, ISS staging, TC classification and time to disease progression (PFS) suggesting that clinical expression of resistance was identical. Both trials contained high dose melphalan as a mainstay in the standard therapy for all patients enrolled.

VAD = Induction with vincristine, adriamycin and dexamethasone. PAD = Induction with bortezomib, adriamycin and dexamethasone. CVAD = Induction with cyclophosphamide, vincristine, doxorubicin and dexamethasone. CTD = Induction with cyclophosphamide, thalidomide and dexamethasone.

In brief, the **HOVON65/GMMG-HD4** trial evaluated the efficacy of bortezomib induction and maintenance therapy in newly diagnosed MM patients eligible for HDM therapy. A total of 827 patients were randomly assigned to induction with vincristine, adriamycin and dexamethasone (VAD) vs. bortezomib, adriamycin and dexamethasone (PAD). Subsequently patients received one or two cycles of HDM 200 mg/m^2^ depending on local policy. Patients assigned to the PAD arm received two years of bortezomib maintenance post-HDM whereas patients randomized to the VAD arm received two years of thalidomide maintenance [16]. The **MRC Myeloma IX** randomized a total of 1960 newly diagnosed MM patients to induction with cyclophosphamide, vincristine, doxorubicin and dexamethasone (CVAD) vs. cyclophosphamide, thalidomide and dexamethasone (CTD) regimen followed by one cycle of HDM 200 mg/m^2^. After, HDM patients were secondarily randomized to thalidomide maintenance vs. no maintenance [17]. Thus, both trials contained HDM as a mainstay in the therapy for all patients enrolled.

Finally, available on-line data was included from 156 patients in the **APEX phase-3 trial** investigating the efficacy of bortezomib vs. high-dose dexamethasone for relapsed MM patients. This trial acted as a negative control cohort in order to exclude the RI predicted response to treatments not containing HDM [18], [19]. Response to treatment was defined primarily by the European Group for Blood and Marrow Transplantation (EBMT) criteria for evaluating disease response and progression in patients treated by high-dose therapy and haemopoietic stem cell transplantation [20], but with the International Myeloma Working Group (IMWG) criteria for very good partial response (VGPR) added [21].

### Myeloma plasma cell purification and gene expression microarray analysis

All included patients had bone marrow plasma cell (PC) enrichment, RNA extraction, cDNA processing, labelling, hybridization and microarray analysis performed as previously described [16], [17]. In brief, bone marrow CD138 positive PCs were enriched by positive magnetic micro beads selection (MACS) (Miltenyi Biotec B.V., Utrecht). Samples were qualified by >80% purity and viability following flow cytometry analysis using the EMN recommendations. RNA was isolated by a DNA/RNA prep kit (Qiagen, Venlo) and concentration measured using the NanoDrop spectrophotometer (Thermo Fisher Scientific). RNA quality and purity were assessed and RNA processing, labelling and hybridization to microarrays were performed at the Erasmus Medical Center, Rotterdam NL [16] or Institute of Cancer Research, London UK [17] and deposited on-line as described below. In the APEX data set myeloma cells were enriched via negative selection using magnetic assisted cell sorting (MACS) and deposited as described [18].

### Microarray data available on line

Details of on-line information used in the present manuscript are described below.

1. The *HOVON65/GMMG-HD4 data* originate from Affymetrix U133 plus 2.0 microarrays. The .CEL files for the gene expression data are available at http://www.ncbi.nlm.nih.gov/geo/ as GEO accession number GSE19784.
2. The *MRC Myeloma IX data* originate from Affymetrix U133 plus 2.0 microarrays. The .CEL files for the gene expression data are available at http://www.ncbi.nlm.nih.gov/geo/ as GEO accession number GSE15695.
3. The *APEX data*, used as the negative control, originate from Affymetrix U133A microarrays and are available at http://www.ncbi.nlm.nih.gov/geo/ as GEO Series Accession Number GSE9782. However, this data is MAS 5.0 normalized, so we obtained access to the original .CEL-files and used them for further analysis [18; Mulligan, Personal Communication).
4. The *B Cell Gene Expression Data* contain .CEL files for the 18 malignant B-cell line microarrays which have been deposited at http://www.ncbi.nlm.nih.gov/geo/ under GEO accession number GSE22759.

### Molecular classification of MM

The pattern of translocations and cyclin D expression (TC) and the University of Arkansas for Medical Sciences (UAMS) risk classification based on Affymetrix gene expression microarray data was done on the available data set according to procedures described by Bergsagel and Shaughnessy and co-workers, respectively [4], [22].

### Strategy for the generation of amelphalan RI

**Figure 1:**
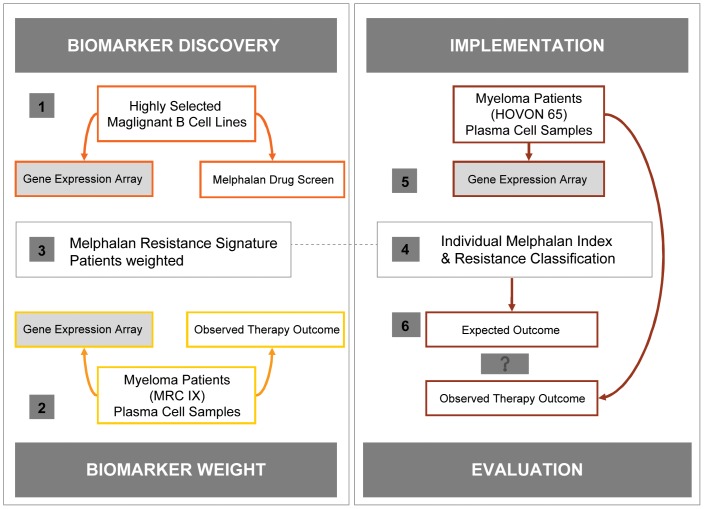
Summary of the stepwise development, adjustment and validation of the resistance gene list. Numbers relate to step 1–6 as illustrated in the figure. 1) First, The analysis starts by identification of candidate biomarkers by a sparse partial least squares algorithm (SPLS) to build a predictive gene list based on correlations with the GI50 values of the cell line panel, regarded as *“biomarker discovery”*. 2) Second, the candidate genes were trained to ensure weighted expression in the myeloma data set from the patient cohort in the clinical trial MRC Myeloma IX, to derive a gene signature model, predictive of resistance to melphalan – a step which is regarded as *biomarker weighting*. The weighting was performed by multivariate Cox regression with PFS as dependent variable and gene expression of the 19 genes as independent variables resulting in a weighted gene signature. 3) The weighted melphalan resistance gene signature is used to define a melphalan RI. 4) The signature is used to classify each tumour from the clinical trial HOVON65/GMMG-HD4 based on the individual GEP data. 5) The RIs were defined to be the linear predictor of the multivariate Cox regression, i.e. calculated for each individual clinical sample by a linear combination of the 19 gene expressions using the weights from the multivariate Cox regression model. 6) Finally, the molecular prediction of resistance to melphalan therapy was compared to the actual observed PFS and OS. – a step regarded as *implementation and evaluation*.

Global GEPs for the 18 B-cell myeloma, plasmacytoma and lymphoma cancer cell lines prior to a melphalan 50% growth inhibition (GI50) screen was conducted as described [11]. The GEP and drug screen were used according to the following work plan as illustrated in Figure 1.

First, a sparse partial least squares algorithm (SPLS) was used to build a predictive melphalan resistant gene list based on the GI50 values of the cell line panel - a step which can be regarded as *biomarker discovery* (Figure 1 left).

Second, the 19 candidate genes were used as predictors in a multivariate Cox regression model with the PFS from the MRC Myeloma IX data as endpoint. The coefficients of the resulting linear predictor were used to form an *in vivo* weighted gene list to derive a gene signature model, predictive of resistance to melphalan – a step which is regarded as *biomarker weighting* (Figure 1 left).

Third, this gene signature was used to assign a melphalan RI for each tumour from the clinical trial HOVON65/GMMG-HD4 based on the individual GEP data (Figure 1 right). The RIs were defined to be the linear predictor of the multivariate Cox regression, i.e. calculated for each individual clinical sample by a linear combination of the 19 gene expressions using the weights from the multivariate Cox regression model.

Finally, the molecular prediction of the expected outcome following melphalan therapy was examined with respect to PFS and OS – a step regarded as *implementation and evaluation* (Figure 1 right).

### Statistical analysis

All statistical analyses were done with R version 2.13.10 [23] and Bioconductor [24]. The .CEL files were background corrected and normalized for each study by the just.rma function from the Affymetrix package. Kaplan-Meier survival analysis, log-rank tests and Cox proportional hazards models were calculated with the built-in R-package survival. A nonlinear relationship between the predicted response to treatment and the RI was noticed and the relationship was estimated by restricted cubic splines (RCS) by means of the R-package Design. The significance level is set to 0.05 and the hazard ratios (HR) are given with 95% confidence intervals. We have calculated time dependent receiver operating characteristics (ROC) curves for cumulative PFS and OS to illustrate sensitivity and specificity of the RIs. ROC curve analysis was included by means of the R package survcomp [25].

## Results

### Comparison of the clinical data sets

The available trial data sets used in the present analysis included patients treated with one or two high doses of melphalan, supported by autologous stem cell transplantation as standard therapy [16], [17]. Comparison of the two independent cohorts for demographic as well as ISS staging did not reveal clinical differences between the trial sets. There was no difference in PFS as given in Table 1, suggesting that clinical expression of resistance was identical.

Patients included in the present analysis all had a bone marrow malignant plasma cell purification step performed for global GEP analysis. Of special interest, no significant difference was observed between the two datasets comparing the TC classification based on the GEP analysis as shown in Table 1, suggesting an identical distribution of biological subgroups defined by early genetic events in pathogenesis.

### Reweight of the melphalan resistance gene list

As illustrated in Figure 1, the first step regarded as “biomarker discovery” included the identification of candidate genes following correlation of gene expression data and melphalan sensitive of the B cell lines [11]. During the next step regarded as “biomarker weight” the resistance gene list was modified by the approach of Lee and co-workers [15] with the aim of adjusting the impact of each gene discovered *in vitro* by the time to disease progression from HDM as an indicator for clinical resistance.

The *in vivo* modified or reweighted gene list from adjustment in the MRC Myeloma IX trial data set is shown in Table 2 and was subsequently used for assignment of an individual melphalan RI for each patient in the HOVON65/GMMG-HD4 data set available for validation as described below.

**Table 2:** Generated probe sets for melphalan resistance weighted and re-weighted.

Probe ID	Gene Symbol	Location	Weight	New Weights
204204_at	SLC31A2	9q31-q32	−0.025	−0.507
219748_at	TREML2	6p21.1	−0.033	−0.38
203708_at	PDE4B	1p31	−0.053	−0.319
201990_s_at	CREBL2	12p13	−0.046	−0.314
219049_at	CSGALNACT1	8p21.3	−0.037	−0.226
218751_s_at	FBXW7	4q31.3	−0.044	−0.184
212055_at	C18orf10	18q12.2	0.025	−0.184
205990_s_at	WNT5A	3p21-p14	−0.065	−0.077
204786_s_at	IFNAR2	21q22.1, 21q22.11	−0.033	−0.0522
217825_s_at	UBE2J1	6q15	−0.02	0.0401
201889_at	FAM3C	7q31	−0.039	0.074
206405_x_at	USP6	17p13	−0.038	0.0888
203895_at	PLCB4	20p12	−0.015	0.0982
212122_at	RHOQ	2p21	−0.016	0.137
205862_at	GREB1	2p25.1	−0.034	0.187
221210_s_at	NPL	1q25	0.032	0.189
213555_at	RWDD2A	6q14.2	−0.019	0.195
217104_at	ST20	15q25.1	0.012	0.201
202043_s_at	SMS	Xp22.1	0.011	0.486

The first four columns contain a list of probes, genes, location and weights for candidate biomarkers obtained by comparing gene expression data between melphalan sensitive and resistant B cell lines (11). In the fifth column the candidate gene list has undergone an *in vivo* disease reweighting by PFS in the MRC Myeloma IX training set.

### The melphalan RI differs between TC classes

**Figure 2:**
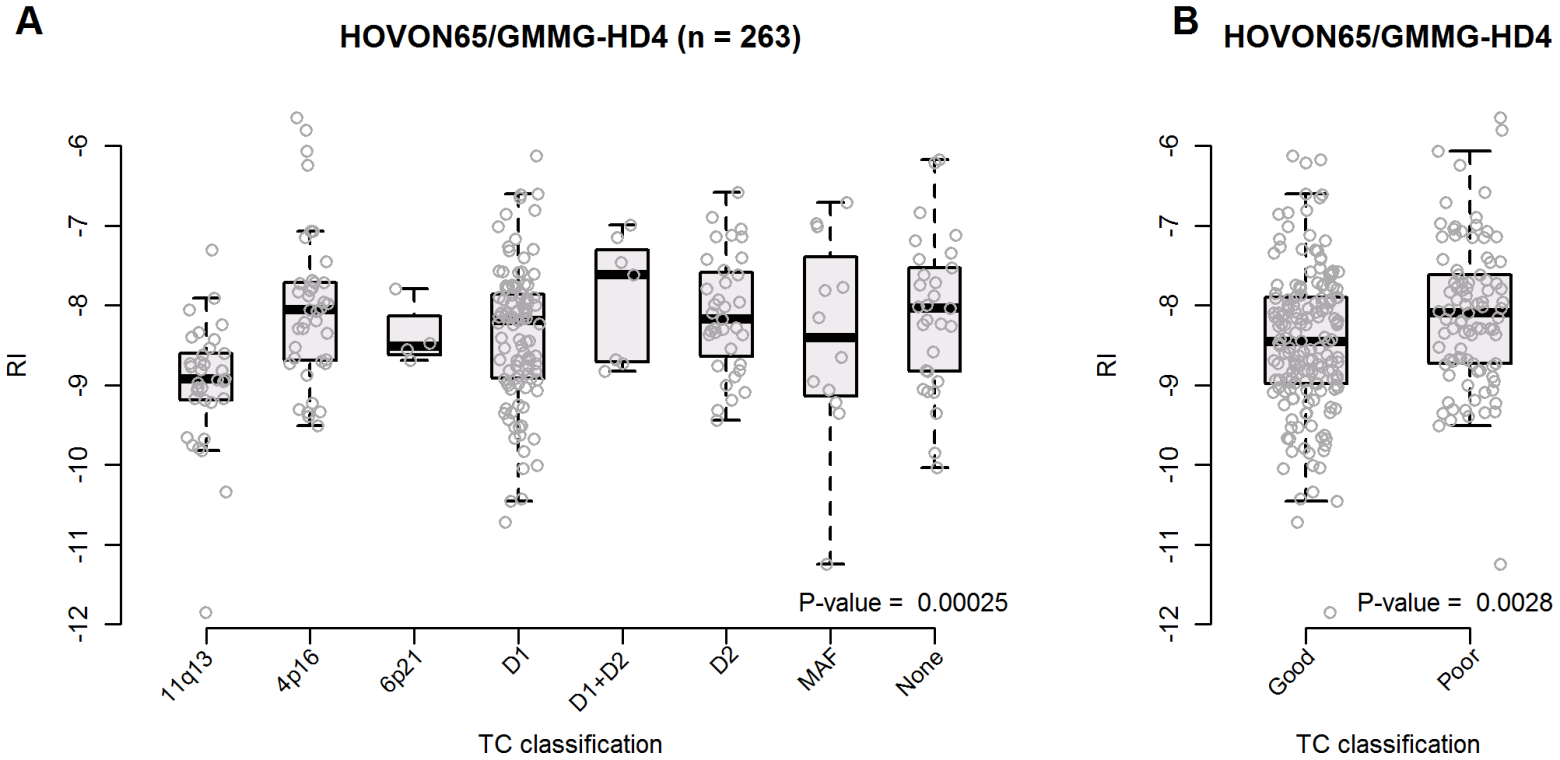
The melphalan RI differ between TC classes. The translocation and cyclin D defined TC classification involving early oncogenic events were applied to the HOVON65/GMMG-HD4 data set and in panel A) each of the 8 classes of tumours showed different melphalan RI levels (P-value  = 0.00025). In panel B) these classes were grouped into two groups with good or poor prognosis with different melphalan RI levels (P-value  = 0.0028).

The biological TC classification defined by early oncogenic events was applied to the HOVON65/GMMG-HD4 data set and each class of tumours compared to the RI assigned level of melphalan resistance. The comparison indicates a difference between the predefined classes ranked from 11q, 6p, MAF, D2, D1, 4p, D1+2 and unclassified as illustrated in Figure 2A. In addition the TC classes could be divided into poor and good risk groups as defined previously [22] and we found these groups had a significantly different RI level (P-value  = 0.0028) as illustrated in Figure 2B.

We also observed varying levels of resistance by comparing the eleven classes defined by the UAMS classification of 8 main subgroups (CD1, CD2, MF, MS, PR, HY, LB, MY) and the 3 additional subgroups (NFKB, CTA, and PRL-3) [22], [16] (results not shown). Finally we compared the RI with the UAMS risk score [26] and documented a significant correlation as illustrated in Figure S1.

### Melphalan RI validation by clinical outcome

**Figure 3:**
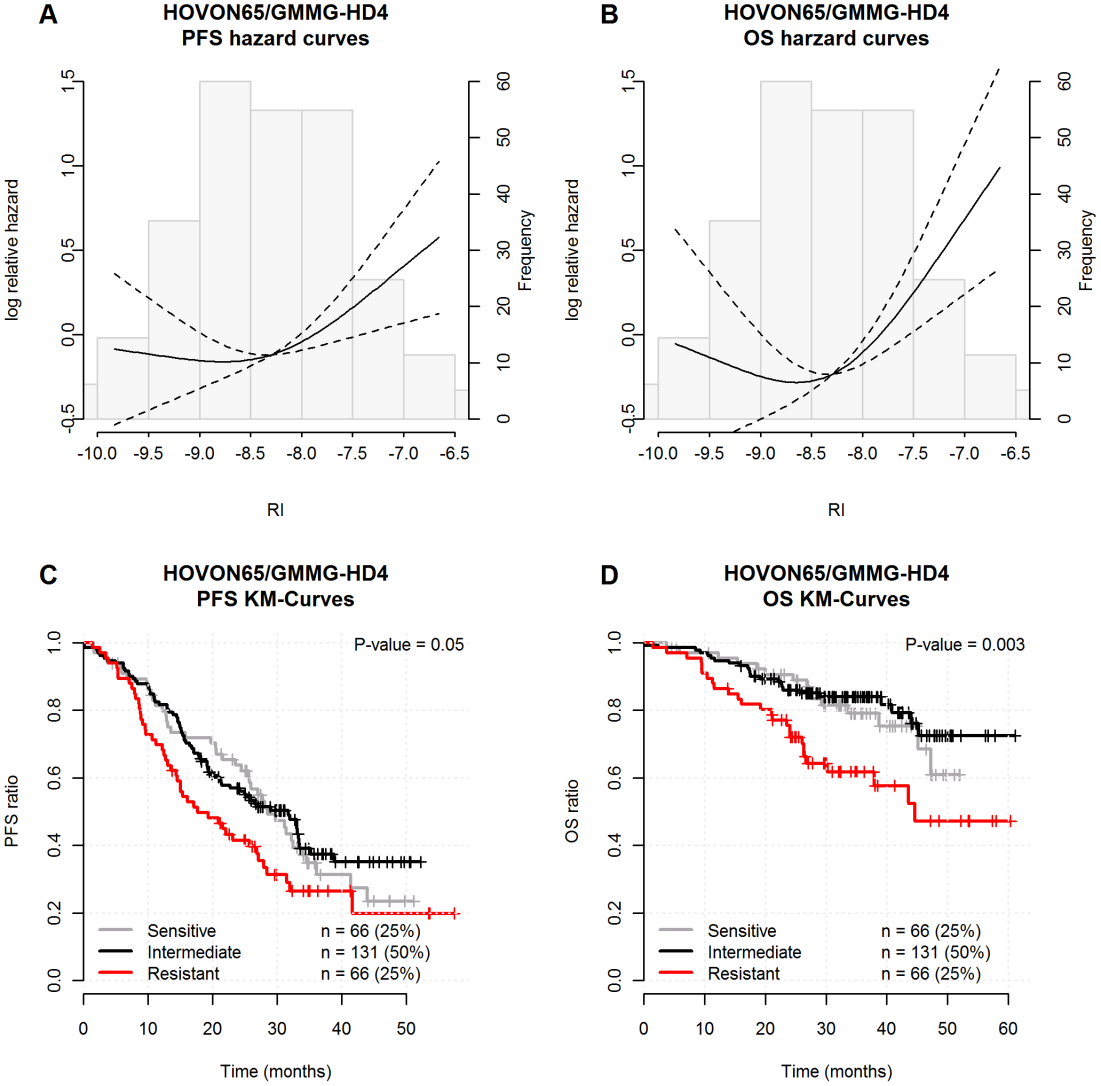
Melphalan resistance gene index validation by clinical outcome. The individual RIs were assigned from gene expression data of the HOVON 65/GMMG-HD4 trial dividing tumor samples into groups of sensitive patients with low 0–25% RI, intermediate RI from 25–75% and resistant patients with the highest 75–100% RI. The impact of this assignment was subsequently evaluated with respect to PFS and overall OS as illustrated by log relative hazard for PFS (1A) and OS (1B) as a function of the individual RI levels. The P-values are the maximum likelihood tests for no RCS-association between log Relative Hazard and the RI and the dashed lines represent 95% confidence intervals. A landmark Kaplan-Meier analysis was performed from the time of HDM and we found that resistant, intermediate and sensitive patient groups had a median PFS of 18, 32 and 28 months, respectively (1C). The OS for the resistant group had a median of 45 months but not reached for the intermediate and resistant groups (1D) following a median observation time of 38 months. The P-values are the log-rank-test results for no difference between the estimated survival curves.

As indicated in Figure 1, we continued with the fourth “implementation step”, where an individual RI was assigned based on each patients' gene expression data in the HOVON 65/GMMG-HD4 trial, dividing tumor samples into groups of sensitive patients with low 0–25% RI, intermediate RI from 25–75% and resistant patients with the highest RI of 75–100%. The impact of this assignment was subsequently evaluated with respects to PFS and OS as illustrated in Figure 3. The log relative hazard curves for PFS and OS, given as a function of the RI, is illustrated by the upper part and Kaplan-Meier survival curves for the assigned groups is illustrated by the lower part. The figure shows that the resistant group (highest RI) has a significant inferior PFS (Figure 3C, P-value  = 0.05) and OS (Figure 3D, P-value  = 0.003) compared to the intermediate and sensitive groups. The present landmark analysis was performed from the time of HDM and we found that resistant, intermediate and sensitive patient groups had a median PFS of 18, 32 and 28 months, respectively. The median OS for the resistant group was 45 months but not reached for the intermediate and resistant groups following a median observation time of 38 months.

**Figure 4:**
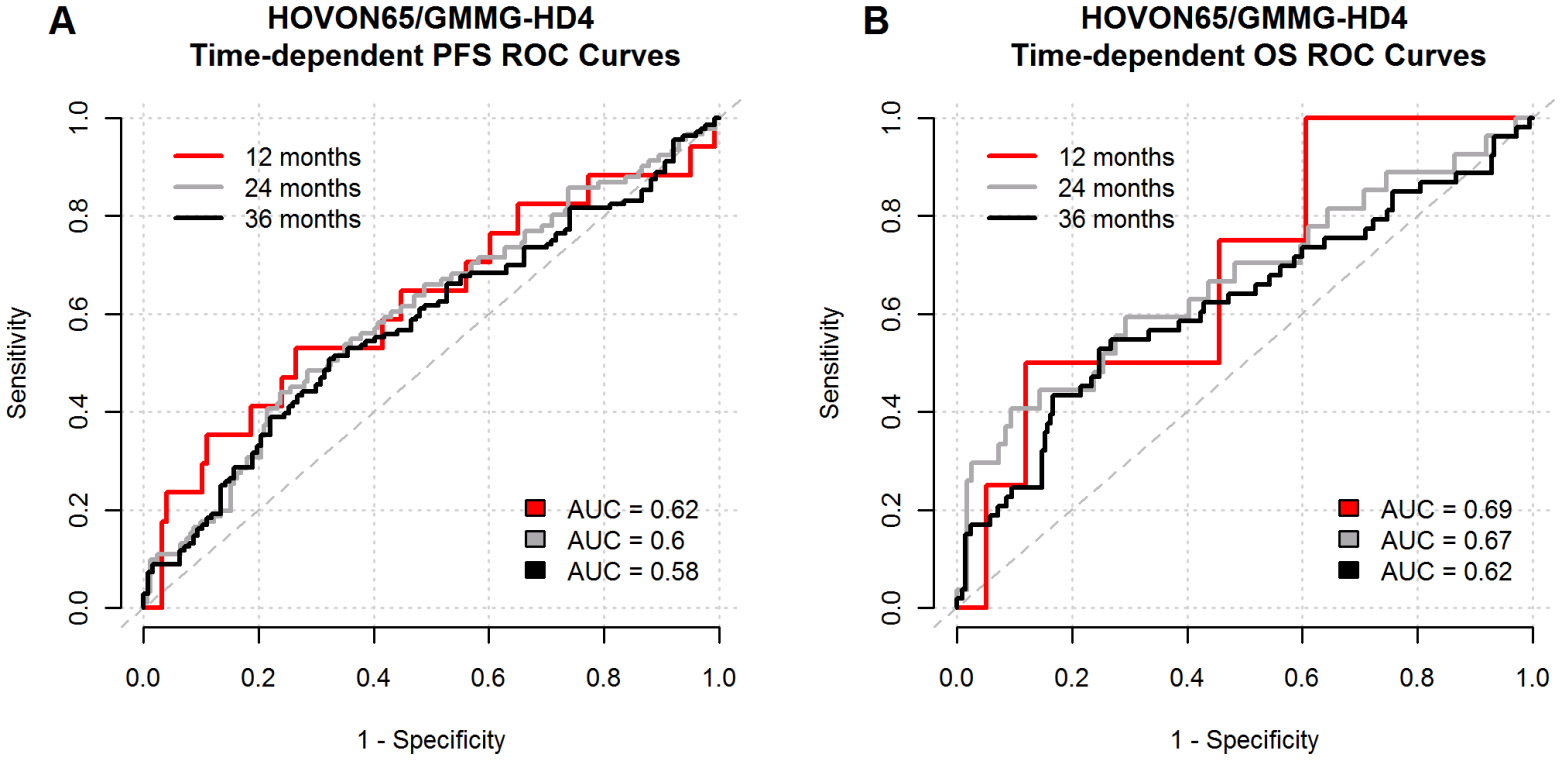
The poor diagnostic accuracy illustrated by ROC curves. The diagnostic accuracy of the RI to predict the PFS and OS was evaluated by ROC curves. The true positive rate (sensitivity) was plotted as a function of the false positive rate (1- specificity) for a series of time dependent cut-off points illustrating the level of discrimination is quantified by area under curve (AUC) for OS and PFS being poor between 60–70%.

The diagnostic accuracy of the RI to predict PFS and OS was evaluated by ROC curves. The sensitivity was plotted as a function of the specificity for a single cut-off point dividing the samples into a sensitive and resistant group. The results illustrate a poor discrimination capacity by the area under curve (AUC) being between 58 and 62% for OS and 62–69% for PFS, as illustrated in Figure 4.

Based on the log-relative HR plots in Figure 3A and 3B, which indicated that the sensitive and intermediate groups have identical resistance profiles, we decided to merge the two groups for further analysis of the clinical impact of the melphalan RI. The merged sensitive group with RI from 0–75% and the resistant group with RI >75% were analysed by univariate (P-value  = 0.003 for PFS and P-value  = 0.00089 for OS) as well as a multivariate Cox proportional hazard models (P-value  = 0.0063 for PFS and P-value  = 0.0025 for OS). This documented an association between the melphalan RI and PFS as well as OS, independent of the ISS staging as shown in Table 3. The best described prognostic and public available gene signatures seem to be from the University of Arkansas for Medical Sciences (UAMS), the Intergroup Francophone du Myeloma (IFM) and the HOVON prognostic classification gene lists [22], [26]. Therefore we compared the melphalan RI with the UAMS risk score documenting a significant correlation as illustrated in Figure S1. This forced us to perform an extended multivariate Cox proportional hazard analysis with the UAMS high risk signature as a variable indicating that the melphalan RI is also independent of this signature for PFS and OS as shown in Table S1. The appropriateness of the Cox proportional hazard models using the dichotomized resistance index were checked using cumulative martingale residuals (data not shown).

**Table 3:** Univariate and multivariate Cox proportional hazard models.

	Univariate			Multivariate		
	Hazard ratio	95%CI	P-value	Hazard ratio	95%CI	P-value
**PFS**						
Age	0.989	(0.97, 1.01)	0.31	0.987	(0.97, 1.01)	0.2
SexFemale	1	-	-	1	-	-
SexMale	1.07	(0.78, 1.48)	0.67	1.06	(0.77, 1.47)	0.71
ISS1	1	-	-	1	-	-
ISS2	1.45	(0.99, 2.14)	0.058	1.43	(0.97, 2.1)	0.069
ISS3	1.97	(1.31, 2.96)	0.0011	1.91	(1.27, 2.88)	0.0019
RI(0,75]	1	-	-	1	-	-
RI(75,100]	1.73	(1.2, 2.47)	0.003	1.65	(1.15, 2.37)	0.0063
**OS**						
Age	0.991	(0.96, 1.02)	0.57	1.17	(0.7, 1.97)	0.55
SexFemale	1	-	-	1	-	-
SexMale	1.28	(0.77, 2.15)	0.34	2.36	(1.16, 4.79)	0.017
ISS1	1	-	-	1	-	-
ISS2	2.35	(1.16, 4.76)	0.018	4.01	(1.98, 8.11)	0.00011
ISS3	4.28	(2.13, 8.63)	4.7e-05	2.28	(1.33, 3.88)	0.0025
RI(0,75]	1	-	-	1	-	-
RI(75,100]	2.45	(1.44, 4.14)	0.00089	2.28	(1.33, 3.88)	0.0025

The sensitive and intermediate RI groups were merged into a common non-resistant group of patients. The redefined non-resistant (RI 0–75%) and resistant (RI 75–100%) groups were analysed by univariate (P-value  = 0.003 for PFS and P-value  = 0.00089 for OS) as well as a multivariate Cox proportional hazard models documenting an association with PFS and OS (P-value of 0.0063 and 0.0025), independent of age, sex and ISS staging. The appropriateness of the Cox proportional hazard models using the dichotomized resistance index was checked using cumulative martingale residuals.

Of importance, we also evaluated the impact of RI assignment on remission status following HDM in the HOVON65/GMMG-HD4 trial which revealed no significant difference with respect to obtained complete remission (CR), very good partial response (VGPR), PR, near complete remission (NCR) and progressive disease (PD) three months post transplant (results not shown).

Finally, in order to document that the reweighted gene list was better than pure chance; we randomly selected 1000 lists of 19 probe sets, reweighted the probe sets in MRC Myeloma IX by multivariate Cox regression and tested the predictive properties in the HOVON65/GMMG-HD4 data set. It turned out that 20 (2.0%) of the 1000 random lists in terms of P-values performed better (P-value lower than 0.003) than the reweighted RI in univariate Cox regression and 19 (1.9%) of the 1000 random lists performed better (P-value lower than 0.0063) with respect to multivariate Cox regression. To test the melphalan specificity of the 2% better performing random lists we used them to predict the melphalan resistance of the cell lines. It turned out that 0 and 2 of the lists had significant correlation with the GI50 value for the univariate and multivariate regressions, respectively, supporting that the reweighted gene list was better than pure chance and that the randomly generated lists with good prognostic performance had no correlation with melphalan resistance.

### Melphalan resistance index in patients not treated with HDM

**Figure 5:**
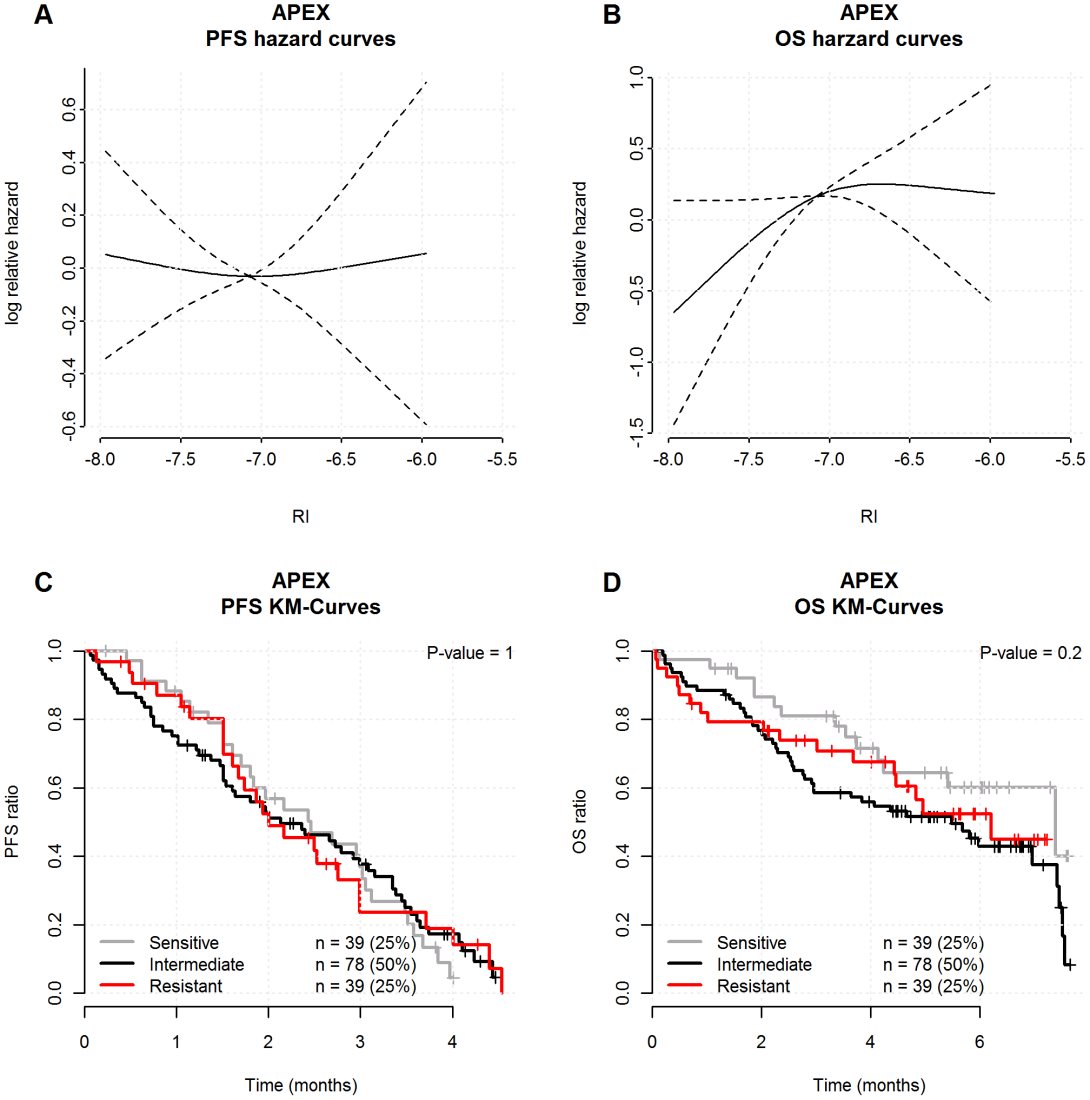
The melphalan RI in patients treated without HDM. Illustration of the negative validation of the approach in a data set from156 relapsed MM patients treated without HDM by inclusion into the APEX trial (18) that compared single-agent bortezomib to high-dose dexamethasone. The individual RIs were assigned from each patients' gene expression data of the APEX trial dividing tumor samples into groups of sensitive patients with the low 0–25% RI, intermediate RI between 25–75% and resistant patients with the highest 75–100% RI. The impact of this assignment was subsequently evaluated with respects to PFS and overall OS as illustrated by log relative hazard for PFS (5A) and OS (5B) as a function of the individual RI levels. The P-values are the maximum likelihood tests for no RCS-association between log Relative Hazard and the RI and the dashed lines represent 95% confidence intervals. A landmark Kaplan-Meier analysis was performed from the time of treatment start which found that resistant, intermediate and sensitive patient groups had no significant differences with respect to the prediction of PFS as well as OS from time of relapse therapy. The P-values are the log-rank-test results for no difference in survival curves.

We also sought to negatively validate the approach in a trial set of data from patients treated without HDM. Therefore we analysed data from 156 relapsed MM patients enrolled into the APEX trial that compared single-agent bortezomib to high-dose dexamethasone [18]. Following melphalan RI classification for each patient we showed no impact in this data set with respect to the prediction of PFS as well as OS from time of relapse therapy (Figure 5).

### Selection of resistance genes for future studies

The resistance gene list comprised 19 genes with corresponding weights and reweights (Table 2). The genes were ranked according to weight, where negative values give the most sensitive marker genes for melphalan and positives values give the most resistance marker genes for melphalan. Interestingly, the list includes the gene *SLC31A2* a membrane pump involved in resistance to alkylating drugs; *FBXW7*, *USP6*, *UBE2*J*1* and *Wnt-5a* involved in ubiquitin proteasome pathways known to affect myeloma biology. Another interesting candidate for further investigation is *CSGALNACT1* which encodes a protein involved in the initial synthesis of chondroitin sulphate, a component of Syndecan-1 (CD138), also known as a central player in multiple myeloma pathogenesis. Further network analysis and functional studies are planned and warranted.

## Discussion

### The concept of the *in vitro* drug screen strategy and its limitations

It is our concept that malignant B-cell lines can be used as a preclinical model, as they have evolved from intrinsic and acquired genetic events in a stepwise process of driver and passenger mutations involved in the molecular mechanisms of resistance. This concept is based on recent work from the Myeloma Stem Cell Network (MSCNET) consortium making well characterized B-cell lines available for further studies, with the limitation that it only detects genetic resistance associated to oncogenesis and does not consider CAMDR or inherited genetic variations also expected to contribute to the resistance phenotype. The availability may accelerate our therapeutic advancements towards individualised therapy as we have recently suggested a list of 19 genes with potential impact on resistance to melphalan [11] following *in vitro* drug screen strategy mimicking the NCI60 cell line based screening platform [12]. Similar studies have been published for cell lines derived from breast and lung cancer [13], [14].

We have now extended this strategy by an *in vivo* modification step including reweight of the resistance gene list in a gene expression data set from the MRC Myeloma IX trial [17] by taking into consideration the grade of clinical resistance by PFS following HDM [15], [27], [28]. We have used this approach to generate a resistance gene index, which we aim to validate in the independent HOVON65/GMMG-HD4 trial [16], by its ability to predict patient outcome by relevant clinical endpoints defining the response to HDM. If successfully validated, this will provide proof of concept to use the panel of malignant B cell lines in a drug screen strategy to identify a range of genes involved in melphalan resistance.

In this report we present results supporting that diagnostic melphalan RI assignments predict PFS and OS in HDM treated patients (Figure 3A–D) – and the melphalan specificity of the RI indicated by analysis of the APEX data, from a clinical trial not including patients treated with HDM during second line therapy (Figure 5A–D). The observation that RI predicted not only PFS but also OS supports that HDM is a cornerstone in the MM therapy, not yet replaced by the new drugs. It must however, be noted that the MM patients in the APEX study were all treated with melphalan during first line induction therapy. The subsequent relapsed disease could thus be interpreted as being caused by melphalan resistant plasma cells that scores high when assigned by the melphalan RI.

One important question in relation to the present work has been the impact of adjustment of the gene list weight to the molecular expression in newly diagnosed tumours. Although this study has validated our RI model it has to be mentioned that the original weighted gene list [11] had a poor performance in the present data sets, with no impact on PFS (P = 0.2) but significant impact in prediction of OS (P = 0.002) in the HOVON65/GMMG-HD4 data set (data not shown). In our mind this observation supports the rationale for adjustment of pure bench generated data by reweighting each gene impact in data sets of newly diagnosed MM patients representing on one hand the biological spectrum of the disease at the time when patients need therapy and on the other hand reduce the influence from late genetic events and the complex genetic picture present in the B-cell lines.

Several other aspects of our current *in vitro* concept need to be studied in the future, first of all the use of inhibition of cell proliferation and growth as an indicator for *in vitro* drug screen outcome. Selection of end points, like grade of apoptosis or estimated number of culture initiating cells, should be considered to improve the preclinical model, depending on the drug in question. Clinically our prediction needs to be studied *in vivo* in relation to known prognostic variables including plasma cell heterogeneity, hyperdiploidy, cytogenetics, gene mapping, methylation mapping, mutations and miRNA assays and of course other biologically or prognostic classification systems and high risk signatures. Finally, the impact of different drug regimens may also be taken into consideration – especially for cross reacting drugs.

A major concern in our validation strategy was that the RI had no impact on remission status 3 months post HDM, indicating that such a validation may need prospective designed studies with focus on exact quantitation of minimal residual disease as proposed by the recent defined stringent CR [21]. It has to be recognized that the remission status post HDM is a difficult end point to evaluate for the effect of HDM as it is also influenced by the induction therapy. Furthermore, CR is a marker of profound tumour reduction depending on the time of follow up and tumour biology as e.g. proliferation status. It has been accepted that CR is a dynamic condition because most CR patients will relapse with time. Finally, we now know from recent studies [37) that patients with standard-risk MM have the same survival regardless of CR status [38), whereas achieving a CR appears to be critical for patients with high-risk disease [39).

Another important objective in the development of our gene list was to demonstrate that the performance of the list is better than pure chance [40, 41). We tested this and noted there is a 2% chance that a random probe set list performs better than the reweighted RI in univariate Cox regression and a 2% chance that a random probe set list perform better with respect to multivariate Cox regression. These findings support that the generated and reweighted gene list was better than pure chance. The 2% random gene lists with better performance and other gene combinations might well have prognostic power but will most probably have no correlation or connection to melphalan specific resistance as it is well-known that many gene lists exist with a spurious correlation with outcome [41]. The latter is also supported as only 0 and 2 gene list predictors of melphalan resistance, identified by uni and mulitvariant cox analysis, were correlated to the GI50 value of the cell lines.

Finally, and most importantly for the clinical limitation of our current findings was the evaluation of the poor diagnostic performance by ROC curves (Figure 4A–B), which concludes that the present output of genes need improvements before clinical implementation. This will include laboratory studies of highly selected genes for *in vitro* function and pathway analysis in order to understand the molecular mechanisms underlying the preparedness to melphalan effect *in vivo*. The ultimate and future goal will be to build a more accurate diagnostic tool by an improved drug screen strategy combined with bioinformatics modelling linking oncogenesis to molecular resistance.

### Resistance and myeloma classification systems

During the last decade more sophisticated and microarray data based classification systems have been described as it was recently reviewed by the International Myeloma Working Group [3]. The original biological classification was based on pathogenetic understanding e.g. hyperdiploid versus non-hyperdiploid or TC classification. One important issue in our work programme was to analyse the level of melphalan resistance in the different biological TC classes. Our present analyses indicate that the melphalan resistances differ between the classes and prognostic groups (Figure 2) indicating that drug resistance in plasma cells may be a consequence of the primary intrinsic events of recurrent gene translocations and cyclin D dysregulation. The prognostic classifications, discriminating outcome in groups of patients treated with multiple drug modalities, based on classical prognostic variables as best illustrated by the ISS prognostic staging system [5]. Here we have documented (Table 3) that the prognosis for patients assigned with a melphalan RI >75% was independent of the ISS prognostic staging system [5]. The best described prognostic gene signatures seem to be from the University of Arkansas for Medical Sciences (UAMS), the Intergroup Francophone du Myeloma (IFM) and the HOVON prognostic classification gene lists [16], [26], [29]–[31] and we extended the multivariate Cox proportional hazard analysis by including the UAMS high risk signature as a variable and showed that the melphalan RI is also independent of this index when considering outcome as PFS and OS (Table S1). Finally and most importantly, the so-called predictive classification should be able to estimate individual outcome of a specific therapeutic intervention and allow for selection and elimination of specific drugs. The UAMS group has reported post drug genomic data identifying patterns associated with drug specific responses [32], [33] and in our ongoing process we have planned pharmacogenetic microdosis studies for specific drugs [34]–[36]. Such data, in parallel with the present programme, are expected to evolve into a useful drug specific and predictive classification system. The range of such a predictive classification system will follow logically from the specific therapy available especially in selection of second line therapy.

### Identification of melphalan resistance gene

One major goal for the present study was to identify genes involved in melphalan resistance. In general, the gene list (Table 2) presents a diverse group of genes involved in numerous key pathways. This indicates that several factors may be involved in determining the level of preparedness of a malignant cell to resist melphalan cytotoxic stress. Of interest in the context of resistance, the gene *SLC31A2* is involved in copper transportation and limited uptake and sensitivity to carboplatin [42], the genes *FBXW7*, *USP6*, and *UBE2*J*1* are involved in ubiquitin regulated pathways [43]–[45] essential in maintaining cellular homeostasis through dynamic switches in protein functions including cell-cycle regulation, proliferation, apoptosis, drug toxicity, and DNA repair and may significantly affect cancer development and the generation of drug resistance [46]. Additionally, the function of Wnt-5a is highly dependent upon ubiquitin proteasome pathways and the gene is active during stem cell growth, cell differentiation and organogenesis and found of biological relevance in MM [47], [48]. A most interesting candidate for further investigation includes *CSGALNACT1* which encodes a protein involved in the synthesis of chondroitin sulphate, a component of Syndecan-1 (CD138) involved in myeloma pathogenesis [49]–[52].

Overall, there is a range of interesting genes to be functionally studied in search of the mechanism(s) involved in both intrinsic and treatment induced resistance to design strategies to overcome it. Such laboratory studies will have benefit from the already available panel of B-cell cancer cell lines defining the preclinical model for malignant B-cell disorders. The ultimate goal is that the programme may lead to the identification of a well documented signature of resistance genes that can “feed forward” into the clinic.

### The main conclusions and future research

This study provides proof of concept to use an *in vitro* drug screen of cancer cell lines for identification of drug resistance genes for further functional analysis and biological behaviour [53], [54]. We expect to identify a range of biological mechanisms involved at different stages of disease progression, thereby making the model useful for better understanding the course of MM. This may in the future help to explain heterogeneity, particularly where there is genetic selection and risk for acquired drug resistance present at relapse or refractory disease [55], [56], with the ultimate goal to improve diagnostic classification and design of mathematical models for evaluation of non-standard personalized medicine [57].

## Supporting Information

Figure S1 **UAMS risk score (RS) and melphalan RI.** Individual correlation between the melphalan RI and the UAMS risk index as defined (26) within the HOVON65/GMMG-HD4 trial data revealed a Pearson correlation coefficient of r = 0.332 (P-value <0.001).
(TIF)

Table S1 **Evaluation of the association between the melphalan RI impact and HDM responses by multivariate Cox proportional hazard models.** The redefined non-resistant (RI 0–74%) and resistant (RI 75–100%) groups were analysed by univariate as well as a multivariate Cox proportional hazard models documenting impact on PFS and OS (P-value of 0.039 and 0.05), independent of age, sex, ISS and the UAMS risk index. The appropriateness of the Cox proportional hazard models using the dichotomized resistance index was checked using cumulative martingale residuals.
(XLSX)
