# Supplementary figures and images for: Proof of the Concept to Use a Malignant B Cell Line Drug Screen Strategy for Identification and Weight of Melphalan Resistance Genes in Multiple Myeloma
Source: PLoS One. 2013 Dec 20;8(12):e83252. doi: 10.1371/journal.pone.0083252 (PMC3869769; doi:10.1371/journal.pone.0083252)

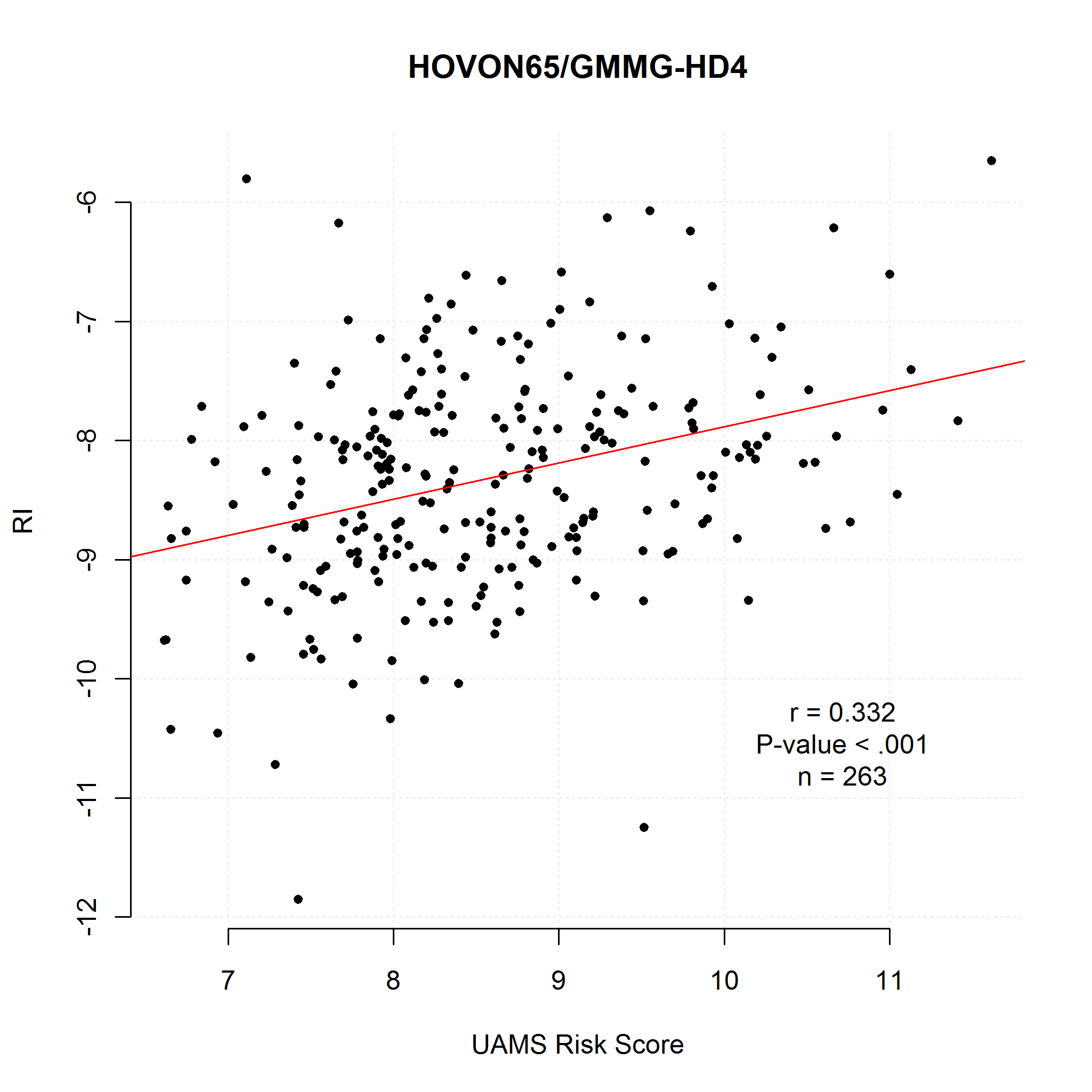

Supplement: Figure S1 — UAMS risk score (RS) and melphalan RI. Individual correlation between the melphalan RI and the UAMS risk index as defined (26) within the HOVON65/GMMG-HD4 trial data revealed a Pearson correlation coefficient of r = 0.332 (P-value <0.001). (TIF) [file pone.0083252.s001.tif]
